# There Is an App for That: Technological Solutionism as COVID-19 Policy in the Global North

**DOI:** 10.1007/978-3-030-65355-2_30

**Published:** 2021-03-20

**Authors:** Linnet Taylor

**Affiliations:** 1 Tilburg University, Tilburg, The Netherlands; 2 Tilburg University, Tilburg, The Netherlands; 3 Tilburg University, Tilburg, The Netherlands; 4 Tilburg University, Tilburg, The Netherlands; Tilburg Institute of Law, Technology and Society, Tilburg Law School, Tilburg, The Netherlands

## Abstract

The COVID-19 pandemic took high-income countries entirely by surprise. Despite funding pandemic preparedness programs in Asia for more than 20 years, donor countries had not experienced an uncontrolled pandemic since HIV in the 1980s. When Ebola, Zika, SARS, and MERS threatened, countries outside the immediate geographic neighborhood or income level of those diseases’ places of origin were left largely untouched. In contrast to the swift, comprehensive response of South-East Asian countries, authorities in Europe and the United States assumed this coronavirus would behave like its predecessors SARS and MERS.

What happened next around the world was both harrowing and illuminating. Lacking protective material resources, the human capacity for contact tracing or understanding of the disease, policymakers in higher-income countries turned to technology for a miracle. The technology sector responded with history’s most extensive hackathon, illuminating the mutual shaping of technology and public health policy. The most striking feature of the technological response to the pandemic has been the degree of what Morozov has called solutionism driving it—the belief that complex problems can be solved by technological intervention alone.

The COVID-19 pandemic took high-income countries entirely by surprise. Despite funding pandemic preparedness programs in Asia for more than 20 years, donor countries had not experienced an uncontrolled pandemic since HIV in the 1980s. When Ebola, Zika, SARS, and MERS threatened, countries outside the immediate geographic neighborhood or income level of those diseases’ places of origin were left largely untouched. This led many northern countries not to take seriously the emergence of the novel coronavirus in 2019 as the existential threat it turned out to be. In contrast to the swift, comprehensive response of South-East Asian countries, authorities in Europe and the United States assumed this coronavirus would behave like its predecessors SARS and MERS, which made their hosts seriously ill and sent them home, reducing their ability to infect others. Instead, the new virus presented itself with a very different epidemiology, hiding mostly unseen amongst the young and active while they infected those around them and ravaging the elderly and sick wherever it spread.

What happened next around the world was both harrowing and illuminating. Lacking protective material resources, the human capacity for contact tracing, or understanding of the disease, policymakers in higher-income countries turned to technology for a miracle. The technology sector responded with history’s most extensive hackathon, illuminating the mutual shaping of technology and public health policy. The most striking feature of the technological response to the pandemic has been the degree of solutionism (Morozov 2013) driving it—the belief that complex problems can be solved by technological intervention alone.

## The Solutionist Approach to the Pandemic

The driving example of solutionism during the first wave of the pandemic was the claim by a group of public health information specialists (Ferretti et al. 2020), that contact-tracing apps were the only way to stop the disease once it had spread out of control. The idea that an app could solve the pandemic was too attractive to ignore, and the policy take-up in higher-income countries was immediate and universal. Existing tracking technologies were repurposed for the pandemic response as vendors took the opportunity of the newly created market to increase the visibility of their products. However, the apps developed soon demonstrated fundamental problems: they were vulnerable to false positives, where people would potentially receive many messages per day from their phone telling them to isolate based on contacts that may not, in fact, have been likely to infect them. Conversely, the apps would also miss many occasions when brief contact did in fact have a high likelihood of resulting in infection—they would be more likely to flag a 10-min conversation with a coronavirus sufferer where both parties were wearing masks than an event where someone was directly sneezed on in a queue or a supermarket aisle.

These problems are compounded by the false negative rate of coronavirus tests (Kucirka et al. 2020). They are further exacerbated by the frequency of asymptomatic infection, where in up to 80% of cases individuals themselves remain unaware they are infectious (Day 2020). In addition to the problem of inaccurate information, apps were not imagined by their advocates as support to human contact tracers, who were instead excluded from the proposed measures almost entirely. Despite evidence from Asian countries that people would only self-isolate based on governmental requests to do so, conveyed by human contact tracers (Bloomberg 2020), most high-income countries decided not to invest in training human contact tracers during the first months of 2020. In the Netherlands, the main infectious disease liaison for the public health authorities, Sjaak de Gouw, played down the value of human contact tracing, calling it pointless while arguing against even testing people for the disease (an essential addition to contact tracing), saying, at the height of the first wave in April 2020: “what is the rationale behind the strategy of extensive testing? You do not prevent infections that way” (NRC 2020a).^1^

With these beliefs driving public health policy advice in the EU, it would have been wise to question the value of an app as the main approach to combating the pandemic. For an app to receive valid information on whether people were infected, testing would have to be both available on demand and accurate enough that people would trust that information rather than facing continual automated demands for quarantine. Moreover, even if an automated system functioned perfectly, the rate of asymptomatic disease meant it would be provided with inadequate data at best (Babones 2020).

Why, then, was the focus so heavily on apps at a time when the remedy appeared to be material resources, behavior change, and building human capacity? Stafford Beer, in his work on organizational cybernetics (Beer 2004), advocated that, in order to analyze a technological system, we should ask what we observe it actually doing in the world, rather than what it is intended to do. If we apply this logic to the case of coronavirus apps in the Netherlands and other EU countries, success on several levels could be observed during the early months of 2020. First, the app development process fulfilled a psychological need for something constructive to do, providing both a goal and a clear deliverable for government and the technical community, at a time when efforts to combat the pandemic were failing and an economy-destroying lockdown was becoming the only option. Second, the process achieved a political function by distracting attention from governmental failures and the resulting death toll (NRC 2020b). Third, they fulfilled a rhetorical function by supporting the public health authorities’ narrative that testing and human contact tracing, which were not possible given the lack of material and trained human resources, were also not necessary (NRC 2020a). However, contact-tracing apps have not, at least so far, fulfilled the need to actually trace the contacts of people infected with COVID-19. As of July 2020, no country has been able to demonstrate that contact-tracing apps can work either in terms of adoption or effective isolation of the infected (Bloomberg 2020).

The app phase of the pandemic response also served a market-building function by orienting at least some of the policy response and public attention away from complex problems of technology provision (ventilators, mask manufacturing capacity, and other material resources) toward the simpler problem of repurposing existing population surveillance infrastructures. Along with the apps, over the first months of the pandemic, many solutionist projects were proposed including systems to detect coronavirus infection from voices (Futurism 2020), detecting infection clusters from sewage analysis using technology originally developed for detecting illegal drug labs (Peccia et al. 2020), and a barrage of biometric systems repurposed from immigration and crowd surveillance toward remote temperature sensing (CSO Online 2020). Some of these efforts are more scientifically credible than others, but all have one feature in common: they do not constitute prevention. Solutionism thrives on desperation, and the coronavirus has, in most of the world, provided a welcoming policy environment for it.

## Lessons of Solutionism

There are higher-level lessons to be drawn from these proposals for pandemic technologies, perhaps most importantly that allowing the market to be the sole driver of technology development will result in technology that primarily serves the market rather than the needs of the public. However, the most relevant lessons for the ongoing effort to combat the pandemic are more immediate. In parallel with the European and US responses to the pandemic, a different set of strategies adopted by South-East Asian countries exists. These, ironically, were based substantially on guidance from institutions founded and funded in the Global North such as the WHO, philanthropies, and universities. They involved early response, immediate travel restrictions, intensive testing and contact tracing, and enforced quarantine where cases were found. Where these measures were taken, both infections and deaths remained strikingly low (BBC 2020; Science 2020). Despite a lack of material and technological resources, at the time of writing in July 2020, Vietnam has reported no COVID-19 deaths, and despite being both dense city-states and international travel hubs, Hong Kong has reported just seven deaths, and Singapore 26 (Worldometer 2020). Meanwhile in the Global North, the problem is already being reframed as technology acceptance and convincing people apps will work (e.g., Metova 2020; Stanford news 2020) although policy attention is, at the time of writing in summer 2020, finally reorienting toward material and human responses to the emergency.

Perhaps the most important lesson we can take with us into the “new normal” of the pandemic from observing technological solutionism in the Global North is this: technology is important to pandemic response, but it must support rather than lead. The practical lessons of the pandemic are ones the North already knew, because it was busy teaching them in the South: the state of the art on how to combat a pandemic is to test, trace, and isolate, and to do this exhaustively and accurately. Yet rather than apply this knowledge, governments turned out to be more ready to listen to Northern innovators than to the public health experts, often internationally funded, in Southern pandemic-affected countries. The response shows that the North has not acquired the necessary capacity for pandemic response, namely flexibility, a focus on the most vulnerable, and human capacity supported by the digital rather than the other way around. Instead, we seem to have trained hard in believing our own rhetoric on technological innovation and the power of the market as solutions to truly existential challenges.

## Footnotes

1 Wat is de rationale achter de strategie van het vele testen? Besmettingen voorkom je er niet mee.
